# Ribosome heterogeneity and specialization of *Plasmodium* parasites

**DOI:** 10.1371/journal.ppat.1011267

**Published:** 2023-04-13

**Authors:** James P. McGee, Jean-Paul Armache, Scott E. Lindner

**Affiliations:** 1 Department of Biochemistry and Molecular Biology, Pennsylvania State University, Pennsylvania, United States of America; 2 Huck Center for Malaria Research, Pennsylvania State University, Pennsylvania, United States of America; 3 Center for Eukaryotic Gene Regulation, Pennsylvania State University, Pennsylvania, United States of America; University of Wisconsin Medical School, UNITED STATES

Malaria remains a major global health burden, causing over 247 million cases and 619,000 deaths in 2021 [1]. This disease is caused by eukaryotic, apicomplexan parasites in the species *Plasmodium*, with the majority of cases caused by *Plasmodium falciparum*. With drug resistance on the rise, it is crucial to identify and exploit specific and essential features of the parasite. One of these differences is the temporally restricted expression of two types of ribosomal RNAs (rRNAs), the Asexual A-type and Sporozoite S-type. These rRNA types are conserved across *Plasmodium* species and have contributed to the reemerging acceptance that ribosomes are heterogeneous and can be specialized in their composition and function in eukaryotes [2,3].

## Historical support for and against ribosome heterogeneity

When ribosomes were first discovered in the 1930s to 1950s, scientists acknowledged that they were heterogeneous, noticing differences in the size and shapes of the particles observed with electron microscopy [4]. This model was furthered by a hypothesis that described how each ribosome would contain the genetic information needed to translate one protein [5]. However, as this hypothesis was disproven and disregarded, so too was the model of ribosome heterogeneity. This shift away from a model of ribosome specialization was supported by the finding that the introduction of foreign bacteriophage RNA into *E*. *coli* was translated by the bacterial ribosomes [6]. The scientific community accepted that ribosomes were nonspecialized machines that would translate any mRNA into protein.

Advances in research methodologies and technologies have enabled more detailed studies of ribosomes, which more clearly showed that ribosomes could be heterogenous in their ribosome protein (RP) composition. These differences in RP composition can be due to the expression of specific RP paralogs in different tissues or organs, such as RPS5A and RPS18A in proliferating tissues of *Arabidopsis* [7] in the sex organs in both *Drosophila* [8] and mice [9], and as cells continue to differentiate and develop [10]. Additionally, in mice, the incorporation of the RP paralog RPL39L (Ribosomal Protein of the Large ribosomal subunit L39-Like) into the ribosome impacts the velocity of translation [11] by changing the polypeptide exit tunnel size and charge, which helps regulate the folding of a subset of essential, male germ cell–specific proteins required for sperm formation [12]. Ribosomes containing RPL10A in developing mice embryos prefer transcripts of members of the canonical Wnt signaling pathway, creating a specialization that is essential for proper mesoderm production during development [13].

Furthermore, while the evolutionarily conserved core rRNA maintains a high degree of conservation across species, it was appreciated that eukaryotes had evolved their rRNA sequences to include expansion segments (ESs). These ESs are protrusions from the core rRNA structure of the ribosome that vary in a species-specific manner [14]. Moreover, ESs have been demonstrated to play regulatory roles, with human ES6S scanning and unwinding mRNA [15], yeast ES27L recruiting methionine amine peptidase (MetAP) to the ribosome [16], and human ES9S recruiting mRNAs with 5′ UTR IRES for cap-independent translation [17]. These latter transcripts were reported to be specific to Hox family mRNAs, but other work has demonstrated these transcripts may not contain an IRES in their 5′ UTR, questioning this potential specialization of ES9S [18]. These findings effectively returned the field to a model of ribosome specialization, with ribosome subunits acting as regulatory units that can be selective for mRNAs and mediate interactions between mRNA and the complete translational machinery through rRNA sequences and structural differences [19].

## Evidence for ribosome specialization in malaria parasites

The sequence heterogeneity and temporal restriction of expression of *Plasmodium* ribosomes lend compelling evidence for the model that *Plasmodium* ribosomes are specialized. *Plasmodium* species commonly contain two types of rRNA, the A-type and the S-type, which vary in their sequence composition and expression patterns. Paralogs of RPs have not been described for *Plasmodium* species, so the current ribosome specialization hypothesis focuses on sequence heterogeneity and temporal expression patterns. *Plasmodium* genomes contain only 3 to 5 copies of rDNA sequences that encode for the 18S-5.8S-28S pre-rRNA, which are located on different chromosomes [2,20,21]. This is in stark contrast to most sequenced eukaryotes, which contain hundreds to thousands of rDNA copies organized in tandem repeats (Table 1) [22]. In this Pearl, we primarily focus on *P*. *falciparum*, which contains five rDNA loci, of which two are nearly identical A-type (A1, A2) and three are S-type (S1 and two copies of S2).

**Table 1:** An overview of key ribosome and rRNA traits across relevant eukaryotes.

Species	*Plasmodium falciparum*	*Plasmodium yoelii and P*. *berghei*	*Babesia microti*	*Cryptosporidium parvum*	*Theileria annulata*	*Toxoplasma gondii*	*Saccharomyces cerevisiae*	*Danio rerio*	*Homo sapiens*	*Triticum aestivum*
**Number of Annotated and Complete rDNA Loci**	5	4	2	2	1	110	Approximately 150	5	Mean of 420 (250–670)	6,650
**Location in Genome**	Chromosomes 1, 5, 7, 11, and 13 contain a single canonical loci	Chromosomes 5, 6, 7, and 12 contain a single canonical loci	Chromosome 3 contains both genomic loci not organized in tandem repeats	Chromosomes 2 and 3 each contain a single canonical loci	Currently unidentified	Chromosome IX	Chromosome 12 contains all loci organized in tandem repeats	Chromosome 4 contains 4 loci: 1 45S-M and 3 undetermined Chr.5 contains 1 loci: 45S-S	Chromosomes 13, 14, 15, 21, and 22 each contain 5 pairs organized in tandem repeats	Chromosomes 6B, 1B, 5D, and 1A contain loci organized in tandem repeats
**Noticeable Differences in Locus Size**	Extended expansion segments; Extended ITS2	Extended expansion segments; Extended ITS2	Lacks an identified 5.8S sequence	N/A	N/A	5S sequence included in the canonical rDNA unit	5S sequence included in the canonical rDNA unit	ITS regions between rRNA types varies; maternal 5S cluster is located in close proximity to the 45S-M locus	Expansion segments are generally longer then most lower level eukaryotes	N/A but plants notably have larger genomes, which correlates with increased number of loci
**Evidence of Restricted Expression Patterns of rRNA**	Yes: Asexual (A) vs. Sporozoite (S) type	Yes: Asexual (A) vs. Sporozoite (S) type	No	No	No	No	No	Yes: 3 types, 1 of which is undetermined. Somatic (45S-S) has ↑rRNA in adult tissues and maternal (45S-M) has ↑rRNA in eggs	No	Yes: different major rDNA loci are expressed in different tissues
**Evidence of Noticeable Attributes of RPs**	No	No	No	No	No	Yes: RPs are up-regulated in extracellular parasites compared to intracellular parasites	Yes: RPs are typically paralogs, so that when a protein is utilized under normal growth conditions its paralog is used during stress conditions	Yes: RPl22 and RPl22l1 antagonistically regulate development	No	Yes: Mitochondrial RPs are differentially regulated during tissue development
**Evidence of Specialized Ribosomes**	Temporal expression pattern coupled with sequence variation most notably at the ESs	Temporal expression pattern coupled with sequence variation most notably at the ESs	No	No	No	No	No	Expression pattern of rRNAs, variation in their sequences and ITS regions, and evidence of RP paralogue function	rDNA contains 470 variant positions; mutations to RPs and paralogs can result in ribosomopathies	No direct evidence in wheat, but in plants, ribosomes play a role in responses to environment and developmental stimuli
**References**	[2,20,39]	[24,33,39]	[39,43,44]	[39,44]	[39,44]	[39,45]	[46]	[47,48]	[49,50]	[51,52,53]

Data are provided for *Plasmodium falciparum*, *Plasmodium yoelii*, *Plasmodium berghei*, *Babesia microti*, *Cryptosporidium parvum*, *Theileria annulata*, *Toxoplasma gondii*, *Saccharomyces cerevisiae* (baker’s yeast), *Danio rerio* (zebrafish), *Homo sapiens* (human), and *Triticum aestivum* (bread wheat). Data sources: [2,20,24,33,39,43–53].

ES, expansion segment; RP, ribosome protein; rRNA, ribosomal RNA.

**Fig 1:**
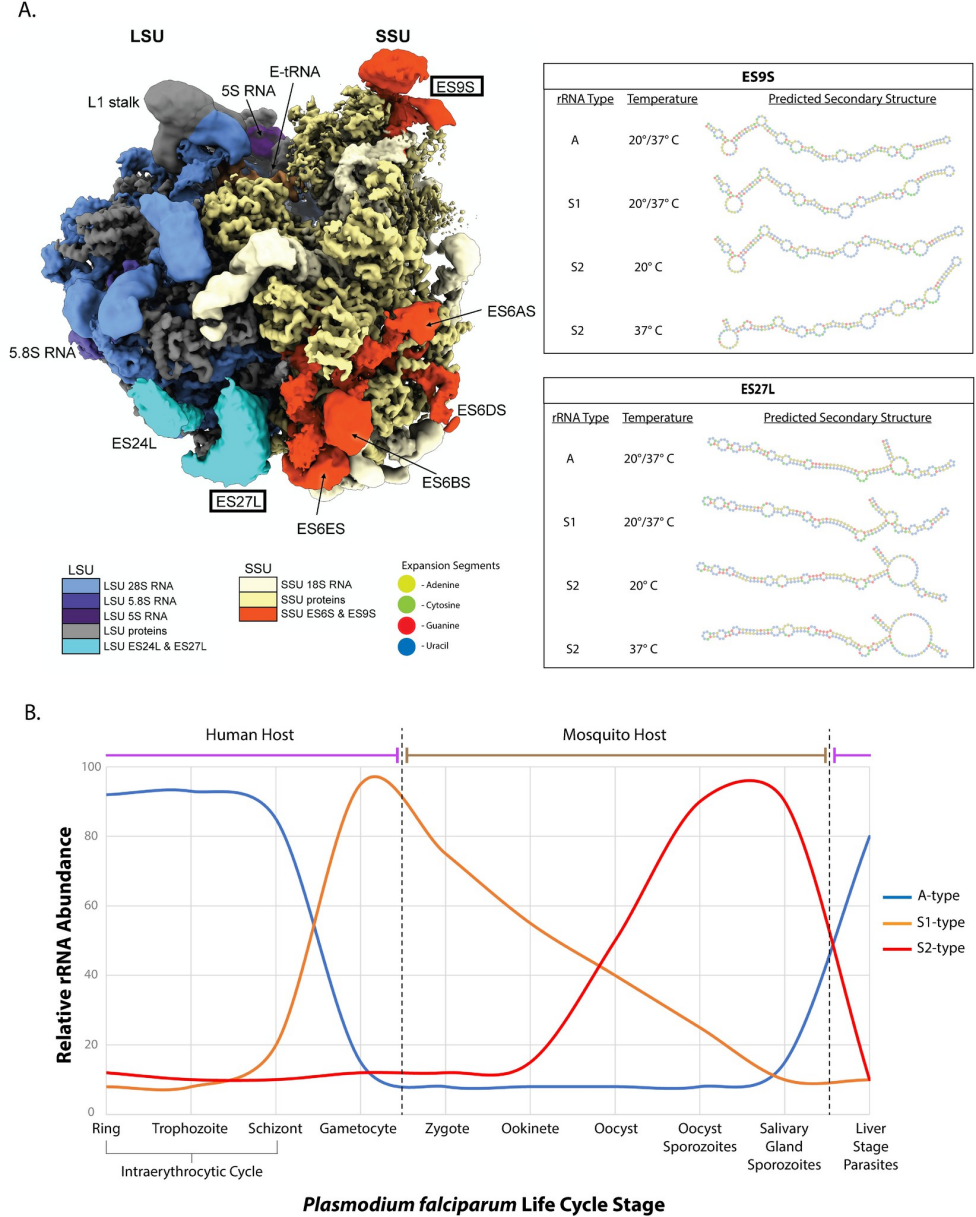
Key features of *P*. *falciparum* ribosomes. (**A**) A model of the Pf80S A-type ribosome was rendered and labeled in ChimeraX using data obtained from previously published structural characterizations [28]. Four ESs are highlighted on the ribosome structure, and the secondary structural predictions of two ESs are shown to represent the variability in the sequences between the *P*. *falciparum* A-type, S1-type, and S2-type rRNA. A-, S1-, and S2-type rRNA sequences were aligned via BLAST [39] to determine sequence differences. Minimal free energy secondary structures of the ES sequences were predicted at 37°C and 20°C using Vienna RNAfold v2.4.18 [40] and were manually base paired at the protrusion location from the core rRNA [28] using the Force-directed RNA (Forna) software [41]. (B) The *P*. *falciparum* rRNA types (A, S1, and S2) were given relative abundance values at each parasite stage to visualize their temporal expression pattern. Relative values are approximated from published data that investigated either rRNA abundance in *P*. *falciparum* [2,30,31] or *P*. *berghei* [33,42] in stages when rRNA abundances in *P*. *falciparum* are not known (e.g., zygote, ookinete, early oocyst, and liver stage parasites). The dashed vertical lines mark transmission events between humans and mosquitoes.

The A1 and A2 rRNAs maintain nearly 100% sequence identity, but variation between the two different S-types is observed across *P*. *falciparum*, *P*. *berghei*, and *P*. *yoelii* species [2,23,24]. In *P*. *falciparum*, the two S2 genes maintain high sequence identity, but a comparison of S2 against S1 shows differences in the ESs in the 28S gene [21]. Furthermore, the A-type rRNA sequence of *P*. *falciparum* varies from the S-type rRNA sequences, primarily at the ESs and, interestingly, also at the highly conserved GTPase center located in the large subunit [2,3,20,25]. In contrast to *P*. *falciparum*, rodent-infectious *P*. *berghei* and *P*. *yoelii* only contain four rDNA loci, two A-type and two S-type. Notably, this variation in ES sequences between the A- and S-type rRNA is conserved across other species, including *P*. *vivax*, *P*. *berghei*, and *P*. *yoelii* [2,3,20,23,24]. These differences result in ESs that differ in their composition and total length and, in some cases, lead to substantial differences in their predicted secondary structures (Fig 1A). These differences in sequence and structure can enable the differential recruitment of effector proteins to ESs on specialized ribosomes [14,16,26]. Despite these biologically interesting differences in rRNA sequences and RP composition, only the structure of the A-type ribosome of *P*. *falciparum* has been characterized via single-particle cryo-EM approaches [27–29] (Fig 1A).

In addition, across *Plasmodium* species, the A-type and S-type rRNAs have different expression patterns that also support a model of ribosome specialization (Fig 1B). In *P*. *falciparum*, an expression pattern was observed using real-time RT-PCR to measure 18S transcript levels specific to the four different rRNA types: A1, A2, S1, and S2 [30,31]. Both A-type rRNAs had their highest abundances during asexual blood stage parasites. Notably, both S1- and S2-type transcripts maintained low (but not negligible) abundances in asexual blood stage parasites [30,31]. In contrast, S1 had the highest abundance during stage III gametocytes, with a gradual decrease in abundance occurring through mosquito stage development, whereas an apparent switch to the S2-type rRNA occurs in oocysts and sporozoites. Importantly, the expression of S1 rRNA in gametocytes validated initial studies via RNA hybridization blotting that probed for either A-type or S-type rRNAs during different life stages, which found the same pattern in both *P*. *falciparum* and *P*. *berghei* [2,20,32]. Overall, across species, A-type rRNA is most abundant during liver and blood stage parasites, with decreasing abundances as the parasite progresses through development within the mosquito. Reciprocally, the S-type rRNAs increase in abundance as the parasite develops within the mosquito and become the most abundant rRNA in salivary gland sporozoites [2,3,33]. Finally, a second switch of rRNA types occurs rapidly after transmission back to a mammalian host early in liver-stage parasites, with the A-type rRNA again becoming dominant in *P*. *berghei* even in the absence of host hepatocytes [34]. This suggests a strong need to switch ribosome types to perform specialized functions when rapid environmental changes and stimuli occur, which would require both selective transcription of the A-type rRNA and targeted decay of only the S-type rRNA. However, it is important to stress that rRNA expression is not strictly stage specific, as A-type rRNA persists in mosquito stages, and S-type rRNA has been detected at lower abundances in blood stages of *P*. *berghei* and *P*. *yoelii* parasites [2,35]. This nonexclusive expression is further supported by single-cell RNA sequencing data that also detected lower abundances of rRNAs at noncanonical stages. For instance, low levels of both S-type rRNAs are present during asexual blood stage *P*. *berghei* parasites but without an increase in abundance in gametocytes [36,37].

## Necessity is the mother of invention (and of specialized ribosomes?)

However, why would *Plasmodium* opt to encode different rRNAs and, moreover, expend great amounts of energy and effort to switch rRNA types? Why is a single type of ribosome insufficient for its needs? Explanations for this have focused on the environmental conditions surrounding the development of malaria parasites in a warm-blooded mammal and a mosquito at ambient temperature. These two host environments differ greatly in both temperature and nutrient availability. To assess if these two parameters dictated rRNA expression, *P*. *falciparum* asexual blood-stage parasites were subjected to different temperatures and glucose concentrations [30,31]. While A1 and A2 rRNA transcripts did not significantly change in abundance with changes in temperature, S1 rRNA transcript abundance slightly increased, and S2 rRNA transcript abundance increased by 15-fold when the temperature was dropped to 26°C [30,31]. To mimic conditions in the mosquito, decreasing glucose concentrations led to an 80% to 85% decrease in A-type rRNA abundance, S1 rRNA levels did not change in abundance, and S2 rRNAs gradually increased in abundance. Furthermore, it was observed that the combination of lower temperature and lower glucose had a synergistic effect that led to a 49-fold increase in S2 rRNA abundance [30,31]. Together, these data suggested that rRNA usage was responsive to environmental changes experienced by *Plasmodium* during its transmission and development, and each might have functions specialized for these life cycle stages.

To date, all reverse genetic studies of *Plasmodium* ribosomes have utilized the rodent malaria species *P*. *berghei* and *P*. *yoelii*. Rapid genome editing has long been possible with both species and has enabled the deletion and disruption of different rRNA sequences to create and phenotype transgenic parasites lacking a particular ribosome component. Genetic modifications of *Plasmodium* are typically made during the asexual blood stage due to technical reasons, so edits to essential genes are limited to those that are not lethal to this stage. Attempts to delete either A-type rRNA gene were noted as being unsuccessful, suggesting that both A-type rRNAs are essential to asexual blood stage parasite development [33]. In contrast, S-type rRNAs in *P*. *berghei* could readily be deleted or disrupted in blood-stage parasites by introducing a deletion cassette into the 18S sequence or by replacing the 28S sequence with a deletion cassette. These deletion lines targeted only one of the two different S-type loci individually, one on chromosome 5 (also called “C”) and one on chromosome 6 (also called “D”). Deletion of either S-type rRNA resulted in a similar number of oocysts compared to the wild type; however, these oocysts were significantly smaller. Despite this, either S-type rRNA deletion could produce sporozoites, which led to the conclusion that S-type rRNAs were interchangeable but were required at sufficient levels to promote proper function. Similarly, a drug-selectable cassette was individually inserted into each of the S-type 18S sequences of *P*. *yoelii* [24]. When the chromosome 5 S-type rRNA was deleted, there was no phenotypic difference compared to wild-type parasites. However, in contrast to the findings with *P*. *berghei*, deletion of the S-type rRNA on chromosome 6 resulted in fewer oocysts per midgut with smaller diameters with no evidence of sporozoite development. This phenotype was partially rescued by plasmid-based expression of the 18S rRNA sequences from chromosome 6, indicating that 18S rRNA sequences provided important functions during mosquito-stage development [24].

## Why are there different types of rRNA in *Plasmodium*?

While this overarching question is not fully answered, current evidence supports the hypothesis that the different types of rRNA of *Plasmodium* are essential and support parasite development within differing host environments. However, the need for any such functional, structural, or specialization differences has yet to be established. This begs the following questions:

1. Do the different rRNA types have roles outside the stages when they are the most abundant?
  The expression profiles of the rRNA types are not black and white, but rather all stages have some degree of expression of all rRNA types. While some of this may be explained by the carryover of a previously expressed rRNA type from one stage to another, such as from blood stage to mosquito stage, the transition from S-type to A-type upon mosquito to mammal transmission occurs rapidly [34]. Moreover, the presence of S-type rRNA in asexual blood stage almost certainly arises from *de novo* transcription. However, interpretations of these observations must factor in precise experimental details, as incomplete separation of stages (e.g., asexual from sexual blood stages) could impact hypotheses of how and why off-stage rRNA transcription occurs. Why then do parasites not suppress transcription or accelerate the decay of the “wrong” rRNA types? An alternate explanation is that these rRNAs are functionally important to the parasite, despite being in low abundance. Answering these questions will require sequential gene editing and precision phenotyping, which is now possible through CRISPR and conditional deletion methodologies.
2. Is the Chromosome 6 (D locus) S-type rRNA just important, or is it truly essential to sporogony?
  The reported inability to delete either A-type locus in blood-stage parasites suggests that both are essential to asexual blood-stage development [33]. However, the essentiality of the S-type rRNAs to rodent malaria parasites during mosquito stage development, more specifically the Chromosome 6 S-type locus, remains unclear. Conflicting phenotypes were observed for *P*. *yoelii and P*. *berghei*, with both exhibiting reduced oocyst sizes, but with either no sporozoites being produced or no defect seen, respectively. Importantly, the essentiality of rRNA types in *P*. *falciparum* has not been determined for any stage, so it is unknown if such an effect is seen in these human pathogens.
3. How are expansion segments used in *Plasmodium*?
  Rapid progress is identifying how and when expansion segments are used for the growth and development of model eukaryotes. Given their importance in attributing specialized functions to ribosomes, we anticipate that novel aspects of translational control will continue to be revealed. With these footholds from other species, it will be exciting to see how *Plasmodium* either adheres to common themes in how each ES is used or how its unique ESs have evolved to drive new interactions and functions. Experiments such as VELCRO-IP developed for yeast may provide clues as to how each expansion segment contributes to translational control [38].
4. Do the rRNA structure and ribosome composition change in mammalian versus mosquito environments?
  The dedication of specific ES sequences to A-type and/or S-type rRNAs may begin to inform us as to why the rRNA types are temporally restricted. In different host temperatures (ambient (mosquito) versus 37°C (mammal)), specific ESs may have different structures and differing abilities to recruit specific effector proteins, which we have predicted here (Fig 1). Together, these ESs could contribute to the folding, composition, and structure of the rRNA and protein components of the ribosome in order to give different ribosome types specialized functions as needed to overcome each host type.
